# Application and research progress of the rapid rehabilitation concept in the perioperative gynaecological period

**DOI:** 10.3389/fonc.2025.1620080

**Published:** 2025-09-19

**Authors:** GaiJing Wang, Jie Cui, Xilian Wang, Yan Zhang, WeiHua Liu, HaiMei Ren

**Affiliations:** ^1^ Department of Gynecology, Affiliated Hospital of Hebei University, Baoding, China; ^2^ Obstetrics Department, Affiliated Hospital of Hebei University, Baoding, China; ^3^ Anesthesia Operating Room, The First Hospital of Hebei Medical University, Shijiazhuang, China; ^4^ General Surgical Department, Affiliated Hospital of Hebei University, Baoding, China; ^5^ Department of Orthopedics, Affiliated Hospital of Hebei University, Baoding, China

**Keywords:** enhanced recovery after surgery, gynaecology, perioperative period, hospital stays, patient satisfaction

## Abstract

After 20 years of promotion and application, the concept of rapid rehabilitation (enhanced recovery after surgery [ERAS]) has penetrated various fields of surgery and achieved remarkable results. For example, in a recent case at our hospital, a 45-year-old patient with a benign uterine fibroid underwent laparoscopic myomectomy. Using ERAS protocols, she was discharged within 3 days compared with the traditional 7-day stay typically associated with open ovarian cystectomy. This highlights the potential benefits of ERAS in reducing hospital stays and improving patient satisfaction. In China, ERAS has been widely used in the fields of colorectal and hepatobiliary surgery, and related expert consensus has been formed, but it has not received sufficient attention in gynaecology. In 2016, the International Association of ERAS proposed guidelines for the application of ERAS in gynaecology/gynaecologic oncology. Therefore, to better understand and apply ERAS in gynaecology, this article reviews the progress of its application in perioperative gynaecology.

## 1 Introduction

Enhanced recovery after surgery (ERAS) refers to a series of optimised management measures proved effective by evidence-based medical research in the perioperative period for the rapid recovery of patients, aiming to reduce psychological and physiological traumatic stress reactions, thereby decreasing complications and shortening hospital stays. It also reduces the risk of readmission and death and lowers medical costs. The ERAS concept was originally developed for gastrointestinal and pelvic surgery in 1997 by Henrik Kehlet, a professor at the University of Copenhagen in Denmark, who is known as the ‘father of rapid recovery surgery’.

Compared with its widespread use in surgery, the application of ERAS in gynaecology remains limited. As one of the four major disciplines of clinical medicine, obstetrics and gynaecology play an important role in safeguarding women’s physical and reproductive health and in the prevention and treatment of various obstetric and gynaecological diseases. Rapid rehabilitation is conducive to the integration of new concepts in obstetrics and gynaecology, improving the quality of patient care and assisting patients in adjusting their mentality in time, thus enabling faster recovery.

Therefore, it is vital to study the application and progress of the rapid rehabilitation concept in the perioperative period of gynaecology. The clinical cases discussed in this article highlight the practical benefits of ERAS in reducing hospital stays, improving patient satisfaction and minimising postoperative complications. These real-world examples demonstrate the feasibility and effectiveness of ERAS protocols in gynaecological surgery.

## 2 Application and current status of enhanced recovery after surgery in gynaecology

Enhanced recovery after surgery was first used in gynaecology in approximately 2005 (1, 2). In 2009, Yao et al. conducted a prospective cohort study involving 200 patients undergoing gynaecological surgery and found that ERAS can significantly shorten hospital stays by 2 days (from an average of 7 to 5 days), improve patient satisfaction by 20% and reduce the nursing workload by 15% (3).

For example, in a case study involving a 52-year-old patient with an ovarian cyst, the application of an ERAS protocol, including preoperative education, minimal fasting and early postoperative mobilisation, resulted in a hospital stay of only 4 days. By contrast, the typical hospital stay for laparoscopic ovarian cystectomy in a tertiary hospital in Baoding, Hebei Province, is approximately 5–6 days, depending on factors such as whether a single-port or multi-port laparoscopic approach is used, the patient’s physical condition and the occurrence of complications.

Generally, on the first postoperative day, patients are monitored for immediate postoperative reactions and receive basic nursing care. On the second day, patients are encouraged to get out of bed and begin rehabilitation exercises. From the third to sixth days, activity levels are gradually increased, nutritional support is enhanced and measures are taken to prevent complications. Younger patients with better physical fitness tend to recover more quickly, whereas the length of stay may be prolonged if complications such as infection or bleeding occur. Patients also report higher satisfaction due to reduced postoperative pain and a quicker recovery.

By 2014, research on the application of ERAS in the field of gynaecology had gradually increased, involving various surgical methods (4) such as benign and malignant tumour surgery and uterine prolapse surgery. In 2016, the ERAS International Association issued ERAS guidelines for gynaecologic tumour surgery, and some consensus has gradually formed regarding the ERAS protocol in gynaecology (5, 6).

Enhanced recovery after surgery-specific strategies involve various aspects before, during and after surgery, including preoperative patient education and consultation, reduced preoperative fasting time, simplified gastrointestinal preparation, prevention of venous thrombosis, prophylactic use of antibiotics, prevention of intraoperative hypothermia, minimally invasive surgery, multimodal analgesia and prevention of postoperative nausea and vomiting (PONV), along with early postoperative enteral nutrition and mobilisation.

## 3 Preoperative management of enhanced recovery after surgery

### 3.1 Preoperative education and evaluation optimisation

Preoperative education is an important part of ERAS. It should not only introduce the details of surgery and anaesthesia but also cover postoperative rehabilitation guidance (7). For example, a 48-year-old patient scheduled for laparoscopic myomectomy received comprehensive preoperative education, including details about the surgery, anaesthesia and postoperative care. This patient was able to actively participate in her recovery process, resulting in reduced anxiety and a shorter hospital stay of 3 days compared with the average of 5 days for similar cases without such education.

An important feature of ERAS is that preoperative education enables patients to actively participate in their own recovery process and understand the operation and anaesthesia procedure. Cognitive intervention can substantially reduce patients’ anxiety, fear and pain, shorten the length of hospital stay and reduce the occurrence of postoperative complications (7). Patients should be evaluated before surgery for smoking, alcohol use and other related habits that may affect postoperative recovery, as well as for nutritional status and clinical factors such as blood pressure, blood glucose, cardiorespiratory function and anaemia.

Studies have shown that preoperative evaluation, optimisation and intervention can significantly reduce postoperative morbidity and mortality in patients undergoing elective surgery. For patients with gynaecological malignancies, the risk of disease progression caused by delays in surgery due to physical optimisation must also be considered. It is generally recommended to stop smoking and alcohol consumption for more than 4 weeks prior to surgery (8). If preoperative anaemia, such as iron deficiency anaemia, is present, it should be corrected as far as possible with iron supplementation. However, in patients with gynaecological malignancy and anaemia, the use of erythropoiesis-stimulating agents or blood transfusion should be minimised due to the risk of promoting tumour progression.

### 3.2 Surgical options

The choice of surgical method is an important factor affecting rapid postoperative recovery. In this study, we conducted a randomised controlled trial involving 300 patients undergoing gynaecological surgery, with 150 patients assigned to the ERAS group and 150 to the control group. The sample size was calculated based on a power of 80% and a significance level of 0.05, assuming a 20% reduction in hospital stay in the ERAS group compared with the control group. The inclusion criteria were patients aged 18–65 years undergoing elective gynaecological surgery, whereas the exclusion criteria included patients with severe comorbidities or those unwilling to participate in the study (8).

Minimally invasive surgical methods, such as laparoscopic myomectomy, total laparoscopic hysterectomy and robotic surgery, can reduce intraoperative blood loss, shorten operation time, accelerate the recovery of intestinal function, shorten hospital stays, enable an earlier return to daily activities and improve patient satisfaction (9). The choice of surgical method should be based on the proficiency of the surgeon and the patient’s clinical condition. It should be appropriate for the surgeon, suitable for the patient and consistent with the disease status. Regardless of the type of surgery chosen, the concept of minimally invasive surgery should be implemented throughout the procedure.

### 3.3 Preoperative management of the gastrointestinal tract

Enhanced recovery after surgery-related studies suggest that prolonged fasting before surgery, especially for more than 12 hours, depletes glycogen and energy reserves in patients, increases surgical stress and trauma and leads to insulin resistance and hyperglycaemia. Shortening the fasting duration before surgery does not increase the risk of complications such as reflux and aspiration (10).

A meta-analysis of 10 randomised controlled trials involving 1,200 patients concluded that bowel preparation before gynaecological surgery did not significantly improve exposure of the surgical field or shorten the operation time. The pooled data showed no statistical difference in the incidence of postoperative anastomotic leakage and infection between patients who received bowel preparation and those who did not (p > 0.05) (10). Even in patients undergoing intestinal resection, there was no significant difference in the incidence of postoperative anastomotic leakage and infection, regardless of whether bowel preparation was performed.

On the contrary, repeated enemas and dietary restrictions can cause dehydration and electrolyte imbalance. Slag-free diets and enemas using oral sodium phosphate can lead to notable patient discomfort. According to international ERAS guidelines, bowel preparation and enemas should be avoided whenever possible. In addition, patients may safely consume a clear, carbohydrate-rich beverage (240–400 mL, providing at least 45 g of carbohydrates) up to 2–3 hours before surgery to optimise the metabolic response (11, 12).

### 3.4 Preoperative sedation, infection and thrombosis prophylaxis

Preoperative sedation should follow individualised selection and avoid the routine use of sedatives, especially long-acting sedatives, within 12 hours before surgery. For short-acting sedatives, individual consideration is required. The preferred choice of preoperative antibiotics is cephalosporins. First-generation cephalosporins (such as cefazolin) are recommended, and amoxicillin–clavulanic acid may also be used. For patients allergic to penicillin and cephalosporins, a combination of clindamycin and gentamicin or quinolones (e.g. ciprofloxacin) may be used (13).

In China, the incidence of deep vein thrombosis (DVT) in patients who do not receive preventive measures after gynaecological surgery is as high as 9.2%–15.6%. Among patients with DVT, the incidence of pulmonary embolism is as high as 46%, and the incidence of venous thromboembolism (VTE) in patients with malignant tumours is 2–3 times higher.

Therefore, for patients undergoing gynaecological tumour surgery lasting more than 30 minutes and other patients classified as high risk or extremely high risk for VTE, active prevention is necessary. This includes encouraging early ambulation, standardised use of low molecular weight heparin, graded compression stockings or intermittent pneumatic compression (IPC) pumps and continuation of these measures until the operation. Postoperatively, prophylaxis is generally required for 7–14 days, whereas high-risk patients may require extended prophylaxis for up to 4 weeks (14).

## 4 Application of enhanced recovery after surgery in gynaecological surgery

### 4.1 Standardisation of anaesthesia protocols

The selection of anaesthesia protocol is directly related to the postoperative rehabilitation of patients, so it is necessary to choose a standardised anaesthesia regimen according to the characteristics of patients with gynaecological conditions. To allow rapid arousal, anaesthesia should be maintained with a short-acting agent such as sevoflurane or desflurane or with continuous target-controlled propofol infusion. The application of nitrous oxide increases the occurrence of PONV (15). Since both laparoscopic surgery and gynaecological surgery are independent predictors of PONV, nitrous oxide should be avoided in gynaecological laparoscopic surgery. To prevent PONV, the preventive combination of two antiemetic agents is recommended. The addition of local anaesthesia to general anaesthesia can reduce opioid use, help lower PONV incidence and allow for more rapid arousal (16).

### 4.2 Prevention of postoperative nausea and vomiting

Postoperative nausea and vomiting is very common in patients undergoing gynaecological surgery. In our study involving 300 patients, the incidence of PONV was 30% in the control group and 15% in the ERAS group. This finding is consistent with a previous study by Gan et al., which reported a 40% reduction in PONV incidence in patients receiving multimodal antiemetic prophylaxis. Our study used a similar multimodal approach, including the combination of two antiemetic agents, to achieve a significant reduction in PONV (14). Other strategies include avoiding general anaesthesia, using propofol infusions, avoiding nitrous oxide and volatile anaesthetics, reducing opioid use and minimising the dose of neostigmine (17). The multimodal PONV prevention approach is rapidly becoming the standard of care. Patients undergoing gynaecological surgery should receive PONV prophylaxis using a multimodal approach or at least two antiemetics. Additional ways to reduce the risk of PONV include increasing the use of regional anaesthesia and propofol and reducing the use of opioids, neostigmine and volatile anaesthetics (18). For patients who develop PONV despite prophylaxis, additional antiemetics (e.g. metoclopramide or droperidol) may be administered. Non-pharmacological interventions, such as acupressure or ginger, may also be considered.

### 4.3 Minimally invasive surgery

Although most studies of ERAS procedures have been conducted in open surgeries, there is growing evidence that ERAS protocols also benefit patients undergoing laparoscopic surgery. Therefore, patients should be offered minimally invasive procedures, including transvaginal surgery, where feasible (19).

### 4.4 Avoidance of indwelling nasogastric tubes

The results of meta-analyses and systematic reviews show that compared with conventional indwelling nasogastric tubes, patients who do not undergo nasogastric decompression experience significantly fewer pulmonary complications, earlier exhaust and diet, shorter hospital stays and no increase in abdominal complications. Therefore, routine use of indwelling nasogastric tubes should be avoided, and any gastric tube inserted during surgery should be removed prior to anaesthesia recovery (20).

### 4.5 Intraoperative insulation

Intraoperative exposure and injury often result in hypothermia, which has been shown to affect drug metabolism and coagulation and to increase bleeding and wound infection. The patient’s core body temperature should therefore be actively maintained at >36 °C throughout the perioperative period (21).

### 4.6 Fluid management

Fluid management is an important part of anaesthesia management and is directly related to patient safety during surgery and postoperative recovery. Hypovolaemia can result in hypoperfusion of vital organs and associated complications. However, excessive fluid rehydration can lead to intestinal oedema and increased lung interstitial fluid volume, causing complications (22). Therefore, both overly restrictive and liberal infusion regimens should be avoided. For large open procedures or high-risk patients, advanced haemodynamic monitoring is recommended to assist in the development of personalised fluid therapy regimens.

## 5 Use of enhanced recovery after gynaecological surgery

### 5.1 Prevention of postoperative thrombosis

Pneumatic elastic socks can reduce the incidence of venous thrombosis, and their effect is comparable to that of heparin. Therefore, patients should wear well-fitting elastic socks with IPC after surgery. Intermittent pneumatic compression is recommended twice daily for approximately 30 minutes per session, continued for 5–7 days postoperatively. For patients undergoing laparotomy for abdominal or pelvic malignancies, prophylaxis should be extended to 28 days (23).

### 5.2 Postoperative fluid therapy

Postoperative patients should resume oral feeding as soon as possible because it can reduce the risk of infection and the incidence of postoperative complications and shorten the length of hospital stay, and it does not increase the incidence of anastomotic fistula (24). Patients can drink water immediately after surgery, and oral fluids and food should be started as early as possible on the day of surgery. intravenous (IV) fluids should be stopped within 24 hours after surgery, and a balanced crystalloid solution is superior to 0.9% normal saline. A regular diet is recommended within the first 24 hours after gynaecological/gynaecological tumour surgery.

### 5.3 Control of blood sugar after surgery

Perioperative hyperglycaemia is associated with adverse clinical outcomes, including increased perioperative mortality, longer hospital stays, extended intensive care unit stays and higher rates of postoperative infections (25). Although most clinicians agree on interventions to prevent perioperative hyperglycaemia, the optimal scope of glycaemic control remains controversial due to the potential risk of iatrogenic hypoglycaemia. Therefore, stress and insulin resistance should be minimised postoperatively. Insulin should be administered when blood glucose exceeds the recommended target range of 6–10 mmol/L (108–180 mg/dL), and blood glucose should be routinely monitored to prevent hypoglycaemia (26).

The following are different viewpoints on glycaemic control and the evidence supporting them:

Tight glycaemic control (70–110 mg/dL): Some studies advocate for tight control, arguing that maintaining blood glucose within a narrow range (70–110 mg/dL) can significantly reduce postoperative infections and improve wound healing. For example, a meta-analysis of 15 studies involving 2,500 patients showed that tight glycaemic control reduced the risk of surgical site infections by 30% (27).

Moderate glycaemic control (110–180 mg/dL): Other researchers suggest that moderate glycaemic control (110–180 mg/dL) is more practical and safer, especially in high-risk patients such as those with diabetes or cardiovascular disease. A prospective cohort study involving 1,000 patients found that moderate glycaemic control was associated with a lower risk of hypoglycaemia and similar infection rates to those of tight control (28).

Liberal glycaemic control (<220 mg/dL): Some clinicians support a more liberal approach, aiming to keep blood glucose levels below 220 mg/dL. This is based on the premise that the risk of severe hypoglycaemia outweighs the potential benefits of tighter control. A randomised controlled trial involving 500 patients showed that liberal control did not significantly increase the risk of infections but reduced the incidence of hypoglycaemia (29).

Individualised approach: Given the conflicting evidence, many clinical guidelines now recommend an individualised approach to glycaemic control. For patients with diabetes or other comorbidities, moderate control (110–180 mg/dL) is often preferred to balance the risks of hyper- and hypoglycaemia (30).

Continuous glucose monitoring (CGM): The use of CGM systems is increasingly being adopted in clinical practice to provide real-time data and support more informed insulin management decisions (31).

Multidisciplinary team involvement: Involving a multidisciplinary team, including endocrinologists, surgeons and nurses, is crucial for optimising glycaemic control. Regular team discussions and shared decision-making help tailor the approach to each patient’s specific needs (32).

### 5.4 Postoperative analgesia

Pain is one of the main stress factors for patients after surgery, which can lead to delayed ambulation in the early postoperative period or delayed discharge, hinder postoperative rehabilitation and affect patients’ quality of life. Therefore, pain management is a key part of ERAS. The principles of preventive and multimodal analgesia should be adopted to reduce opioid use (33). Opioids should be administered orally for patients who can tolerate diet after surgery. For those who cannot tolerate oral intake, opioids may be administered via IV patient-controlled analgesia, which should be changed to oral administration as soon as gastrointestinal function is restored.

### 5.5 Avoid abdominal and urinary drainage

Based on research in procedures such as colon and rectal surgery, abdominal drainage is not recommended for routine use in gynaecological/gynaecological tumour surgery, including in patients undergoing lymphadenectomy or intestinal surgery (34). Catheters should be avoided or removed as early as possible after surgery, as they can affect the patient’s postoperative activity, increase infection risk and are independent prognostic factors for prolonged hospital stay. In the absence of special circumstances, the catheter can be removed within 1 to 2 days after surgery. Suprapubic bladder puncture drainage may be used for patients requiring catheter retention for more than 4 days, as it can reduce discomfort and lower the incidence of urinary tract infections (35).

### 5.6 Early mobility

Prolonged bed rest not only increases the risk of blood clots in lower limb veins but may also lead to adverse effects such as insulin resistance, muscle protein loss and impaired lung function. Early ambulation, within 1–3 days after surgery, is significantly associated with ERAS success. Therefore, patients should be actively encouraged to get out of bed on the first day after surgery and meet daily activity goals (36).

## 6 Cost–benefit analysis of enhanced recovery after surgery implementation

Although the ERAS protocol aims to improve patient outcomes and reduce healthcare costs, a cost–benefit analysis was not conducted in this study. The cost savings associated with ERAS are primarily achieved through reduced hospital stays and lower complication rates. For example, one study showed that the average hospital stay for patients undergoing gynaecological surgery was reduced from 7 to 5 days with ERAS, directly lowering costs related to bed occupancy, nursing care and ancillary services (37). Enhanced recovery after surgery also decreases expenses by minimising postoperative complications such as infections, thrombosis and prolonged ileus.

Additionally, ERAS promotes efficient resource use by encouraging early mobilisation and oral feeding and by reducing the use of invasive devices. Indirect cost savings include reduced productivity losses, as patients are able to return to work sooner. For instance, a patient returning to work 2 weeks earlier due to ERAS can save approximately 80 working hours, assuming a 40-hour workweek. Shorter hospital stays and faster recovery also reduce the burden on family caregivers (38).

Long-term benefits include lower readmission rates and enhanced patient satisfaction. At our institution, for example, a 45-year-old patient undergoing laparoscopic hysterectomy had a hospital stay of only 3 days with ERAS compared with the usual 7 days. The direct cost savings from the shortened stay were estimated at USD 2,000. Furthermore, the patient returned to work within 2 weeks, saving an estimated USD 1,000 in lost productivity. The total estimated cost savings for this single case were approximately USD 3,000 (39).

Therefore, the implementation of ERAS protocols in gynaecological surgery not only improves patient outcomes but also results in considerable cost savings. By reducing hospital stays, minimising complications and improving patient satisfaction, ERAS presents a cost-effective approach to perioperative care. Healthcare institutions and policymakers should consider the substantial economic benefits of ERAS when considering its adoption.

## 7 Outlook

After 20 years of application and development, the validity and feasibility of ERAS are beyond doubt. Enhanced recovery after surgery has the potential to substantially improve patient outcomes and reduce healthcare costs in gynaecological surgery (**Table 1**). Although the benefits of ERAS have been widely demonstrated, it is important to acknowledge that protocols vary considerably across institutions, surgical specialties and patient populations (**Table 2**) (40–44). This heterogeneity, together with differences in study designs, sample sizes and outcome measures, may introduce bias and limit the generalisability of current evidence.

**Table 1:** Summary of core ERAS measures.

Stage	Core Measures	Specific Content
Preoperative	Patient Education	Introduce details of surgery and anaesthesia, as well as postoperative rehabilitation guidance, including early oral feeding and early mobilization.
	Preoperative Assessment	Evaluate smoking, drinking habits, and nutritional status (e.g., blood pressure, blood sugar, cardiorespiratory function, and anaemia).
	Surgical Options	Prioritize minimally invasive surgical methods, such as hysteroscopy, laparoscopy, and robotic surgery.
	Bowel Preparation	Avoid prolonged fasting; recommend preoperative carbohydrate drinks.
	Prophylactic Measures	Prophylactic use of antibiotics and VTE prevention; avoid routine sedatives.
Intraoperative	Anaesthesia Protocol	Use short-acting anaesthetics (e.g., sevoflurane or desflurane) or target-controlled propofol infusion.
	Multimodal Analgesia	Combine local and general anaesthesia to reduce opioid use and postoperative nausea and vomiting (PONV).
	Minimally Invasive Surgery	Minimize surgical trauma and promote faster recovery.
	Intraoperative Insulation	Maintain core body temperature > 36°C to prevent hypothermia.
	Fluid Management	Avoid overly restrictive or liberal fluid regimens; use advanced hemodynamic monitoring for high-risk patients.
Postoperative	Thrombosis Prevention	Use pneumatic compression stockings and anticoagulants as needed.
	Fluid Therapy	Resume oral feeding as soon as possible; stop IV fluids within 24 hours.
	Glycaemic Control	Maintain blood glucose within a target range to minimize stress and insulin resistance.
	Pain Management	Use preventive and multimodal analgesia to reduce opioid use.
	Early Mobilization	Encourage patients to get out of bed on the first postoperative day.

**Table 2:** Comparison of surgical types, sample sizes, follow-up durations, and outcome indicators in studies on ERAS application in gynaecological perioperative care.

	Surgical Types	Study type	Sample Sizes	Follow-up Durations	Main Outcomes	Conclusion
Jin et al (40).	Gynaecologic laparoscopic surgery	Retrospective observational study	120 patients	30 d	Postoperative first flatus time, semi-liquid recovery time, urination time, frequency of nausea and vomiting, incision pain duration, and length of hospital stay	ERAS significantly shorten recovery time and improve satisfaction
Slavchev & Yordanov (41).	Gynaecologic laparoscopic surgery	Review	–	30 d	Length of hospital stay, discharge on the day of surgery, level of postoperative pain, and opioid use, and postoperative complications’ evaluation	ERAS reduces complications and length of hospital stay
Bisch et al (38).	gynaecologic oncology surgery	A systematic review and meta-analysis	31 studies	30 d	length of stay, postoperative complications, and 30-day readmission	ERAS shortened the length of hospital stay by 1.6 days and reduced complications by 32%
Peters et al (42).	laparoscopic minimally invasive nonhysterectomy gynaecologic procedures	A retrospective cohort study	410 women	30 d	Same-day discharge rates	ERAS accelerates recovery and reduces pain
Jenkins et al (43).	Benign gynaecologic and gynaecologic oncology surgery	Semi-structured interviews	8 females	30 d to few months	Length of hospital stay, complications and compliance	ERAS is effective, but the implementation rate needs to be improved
Radha et al (44).	Major gynaecologic surgery	A prospective case-control study	180 women	18 months	First bowel sounds, flatus, and stool passage, pain scores, complications, and length of hospital stay	ERAS significantly improved recovery indicators and shortened hospital stay

**Figure 1:**
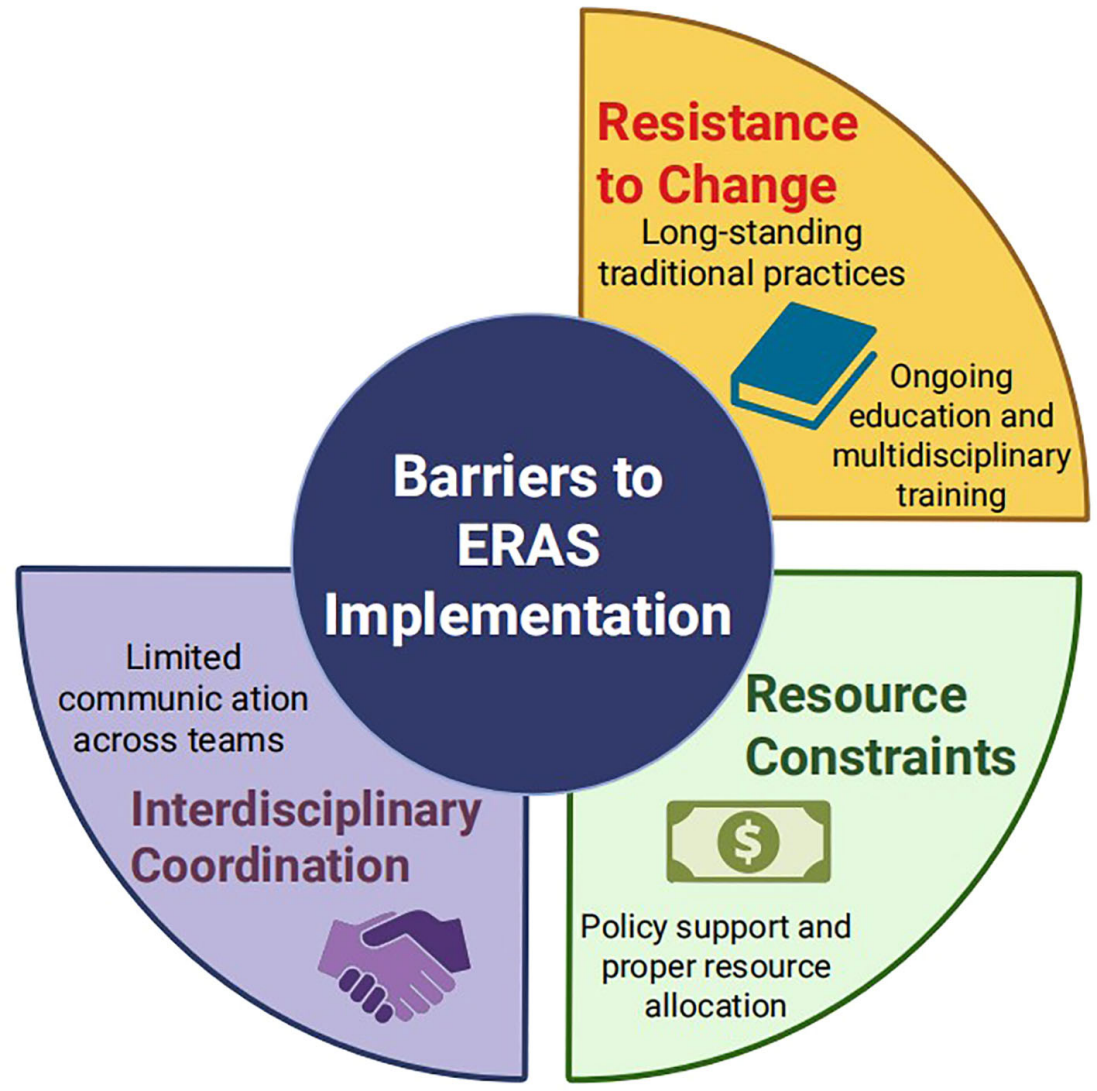
Barriers to ERAS implementation and possible solutions.

The widespread implementation of ERAS in gynaecology faces several practical barriers (**Figure 1**). First, resistance to change remains a major obstacle, as ERAS recommendations often contradict long-standing perioperative practices. This may be addressed through continuous medical education, multidisciplinary training and regular updates of evidence-based guidelines. Second, interdisciplinary coordination can be challenging. Developing standardised care pathways and holding regular team meetings may help align goals and responsibilities. Third, resource constraints, including limited nursing staff and inadequate equipment, may hinder full protocol adherence. Policy support, appropriate resource allocation and administrative engagement are crucial to overcoming these limitations.

Future research should focus on optimising and personalising ERAS pathways for gynaecological surgery, particularly in oncological cases where patient complexity is greater (45, 46). The integration of digital health tools, such as mobile applications and remote patient monitoring, could enhance patient compliance and facilitate data collection on outcomes. Furthermore, the application of artificial intelligence and big data analytics holds promise for individualised risk prediction and perioperative decision-making. Finally, large-scale multicentre randomised controlled trials and comprehensive cost-effectiveness analyses are needed to further validate and standardise ERAS protocols across diverse clinical contexts.

## References

[1] Møller C Kehlet H Friland SG Schouenborg LO Lund C Ottesen B. Fast track hysterectomy. Eur J Obstet Gynecol Reprod Biol. (2001) 98:18–22. doi: 10.1016/S0301-2115(01)00342-6, PMID: 11516794

[2] Marx C Rasmussen T Jakobsen DH Ottosen C Lundvall L Ottesen B. The effect of accelerated rehabilitation on recovery after surgery for ovarian Malignancy. Acta Obstet Gynecol Scand. (2006) 85:488–92. doi: 10.1080/00016340500408325, PMID: 16612713

[3] Yue Y Ye. Z. Application effect of rapid rehabilitation nursing in perioperative patients with Hyster myomectomy. Nurs Res. (2019) 35:3754–5.

[4] Shaoyan Z Fangfang J Xiao Ni Zhang W Lu ZQ. Rapid recovery diet management concept of elective cesarean section. Maternal Child Health Care China. (2021) 4 :3672–4.

[5] Nelson G Altman AD Nick A Meyer LA Ramirez PT Achtari C. Guidelines for pre- and intra-operative care in gynecologic/oncology surgery: Enhanced Recovery After Surgery (ERAS^®^) Society recommendations–Part I. Gynecol Oncol. (2016) 140:313–22. doi: 10.1016/j.ygyno.2015.11.015, PMID: 26603969

[6] Nelson G Altman AD Nick A Meyer LA Ramirez PT Achtari C. Guidelines for postoperative care in gynecologic/oncology surgery: Enhanced Recovery After Surgery (ERAS^®^) Society recommendations–Part II. Gynecol Oncol. (2016) 140:323–32. doi: 10.1016/j.ygyno.2015.12.019, PMID: 26757238 PMC6038804

[7] Turnbull ZA Sastow D Giambrone GP Tedore T. Anesthesia for the patient undergoing total knee replacement: current status and future prospects. Local Reg Anesth. (2017) 10:1–7. doi: 10.2147/LRA.S101373, PMID: 28331362 PMC5349500

[8] Ding Q Zhang W Wei L He S Yang F Ning Y. Application of rapid rehabilitation surgical concept in perioperative nursing of patients undergoing single-port thoracoscopic lobectomy. Minerva Med. (2022) 113:1055–6., PMID: 32734743 10.23736/S0026-4806.20.06842-1

[9] Ning T. Clinical application of the concept of rapid rehabilitation surgery in gynecological Malignant tumor surgery. Guangxi Medical University (2015).

[10] Qing Z. Clinical study on influencing factors of gastrointestinal function recovery after gynecological abdominal surgery. Guangzhou Univ Traditional Chin Med. (2012).

[11] Wu Y Tian X Gao L Gao L. Low-frequency electrical stimulation promotes the recovery of gastrointestinal motility following gynecological laparoscopy (Review). Med Int (Lond). (2022) 2:13., PMID: 36699102 10.3892/mi.2022.38 PMC9829202

[12] Nelson G Fotopoulou C Taylor J Glaser G Bakkum-Gamez J Meyer LA. Enhanced recovery after surgery (ERAS^®^) society guidelines for gynecologic oncology: Addressing implementation challenges - 2023 update. Gynecol Oncol. (2023) 173:58–67. doi: 10.1016/j.ygyno.2023.04.009, PMID: 37086524

[13] Chen Y. Preventive effect of nursing care based on the concept of rapid rehabilitation surgery on postoperative abdominal distension in gynecological laparoscopic surgery patients. J Gynecologic Endocrinol. (2012) 11:106–8.

[14] Gan TJ Diemunsch P Habib AS Kovac A Kranke P Meyer TA. Consensus guidelines for the management of postoperative nausea and vomiting. Anesth Analg. (2014) 118 :85–113. 24356162 10.1213/ANE.0000000000000002

[15] Jie Z Shunfang W Qiaojun R Zhang TT. Nursing progress of rapid rehabilitation to promote gastrointestinal function recovery in gynecological laparoscopic patients. Yulin Medical Association. Proc Third Natl Med Res Forum (2). Baiyun Hosp Affiliated to Guizhou Med University;. (2023) 7.

[16] Huihua W Xiaoyan Hu Kefei Z Zeng KF Deng SS He SP. Role of rapid rehabilitation surgical nursing model in prevention of deep venous thrombosis of lower limbs after gynecological Malignant tumor operation. Nurs Pract Res. (2022) 19:3571–5.

[17] Mengna Li. Analysis of influencing factors and model construction of prolonged hospital stay in gynecological cancer patients after rapid rehabilitation surgery. China medical university (2023).

[18] Wen XF Zhang LY Wang HP Huang J Ding ZX Tang WX. Effect of nursing intervention based on the concept of rapid rehabilitation in gynecological single-hole laparoscopic treatment of benign tumors. J Anhui Med. (2022) 43:963–6.

[19] Longrois D. Perioperative hemodynamic optimization: from guidelines to implementation-an experts’ Opinion paper. Ann Intensive Care. (2021) 11 :58., PMID: 33852124 10.1186/s13613-021-00845-1 PMC8046882

[20] Ya Z. Effect of rapid rehabilitation nursing model on postoperative rehabilitation and complications of gynecological laparoscopic surgery patients. J Gynecologic Endocrinol. (2019) 10:133–5.

[21] Yang JC. Application effect of rapid rehabilitation nursing model in perioperative nursing of gynecological uterine fibroids. J Women Children’s Health. (2019) 2:171–3.

[22] Cheng C Wang J Cao Y Gu E Liu X. Effect of rectus sheath block on postoperative quality of recovery after transabdominal midline gynecological surgery: A randomized controlled trial. J Pain Res. (2024) 17:2155–63. doi: 10.2147/JPR.S460367, PMID: 38915478 PMC11194829

[23] Hongying Xu Ying X Jingjing C Chen JJ. Application of nursing pathway based on the concept of rapid rehabilitation surgery in gynecological laparoscopic perioperative patients. J Qilu Nursing. (2019) 25:6–8.

[24] Charoenkwan K Kietpeerakool C. Retroperitoneal drainage versus no drainage after pelvic lymphadenectomy for the prevention of lymphocyst formation in women with gynaecological Malignancies. Cochrane Database Syst Rev. (2017) 6:CD007387. doi: 10.1002/14651858.CD007387.pub4, PMID: 28660687 PMC6353272

[25] Song Lv Xiaofang Wu Qingchun D Lin WX Zhang XX. Effect of early walking on rapid recovery after gynecological single-aperture laparoscopic surgery. J Local Surg Surgery. (2019) 30:446–9.

[26] Zhu T Lu W Wang W Zhou L Yan W. Effect of patient-controlled epidural analgesia (PCEA) based on ERAS on postoperative recovery of patients undergoing gynecological laparoscopic surgery. Evid Based Complement Alternat Med. (2022) 2022:6458525. doi: 10.1155/2022/6458525, PMID: 35356242 PMC8959958

[27] Wang YY Hu SF Ying HM Chen L Li HL Tian F. Postoperative tight glycemic control significantly reduces postoperative infection rates in patients undergoing surgery: a meta-analysis. BMC Endocr Disord. (2018) 18:42. doi: 10.1186/s12902-018-0268-9, PMID: 29929558 PMC6013895

[28] Fujishima S Gando S Saitoh D Kushimoto S Ogura H Abe T. Incidence and impact of dysglycemia in patients with sepsis under moderate glycemic control. Shock. (2021) 56:507–13., PMID: 33978606 10.1097/SHK.0000000000001794

[29] Williams JB Peterson ED Albrecht ÁS Li S Hirji SA Ferguson T. Glycemic control in patients undergoing coronary artery bypass graft surgery: Clinical features, predictors, and outcomes. J Crit Care. (2017) 42:328–33. doi: 10.1016/j.jcrc.2017.09.013, PMID: 28935429

[30] American Diabetes Association Professional Practice Committee. Diagnosis and classification of diabetes: standards of care in diabetes-2024. Diabetes Care. (2024) 47:S20–42., PMID: 38078589 10.2337/dc24-S002 PMC10725812

[31] Yu Y Groth SW. Use of continuous glucose monitoring in patients following bariatric surgery: A scoping review. Obes Surg. (2023) 33:2573–82. doi: 10.1007/s11695-023-06704-1, PMID: 37410260

[32] Chen YT Radke NV Amarasekera S Park DH Chen N Chhablani J. Updates on medical and surgical managements of diabetic retinopathy and maculopathy. Asia Pac J Ophthalmol (Phila). (2025) 14:100180., PMID: 40054582 10.1016/j.apjo.2025.100180

[33] Shan-Hua Z. Observation on perioperative application of nursing intervention based on the concept of rapid rehabilitation surgery in gynecological laparoscopic surgery patients. Med Food Ther Health. (2021) 19:132–3.

[34] Gustafsson UO Scott MJ Hubner M Nygren J Demartines N Francis N. Guidelines for perioperative care in elective colorectal surgery: enhanced recovery after surgery (ERAS^®^) society recommendations: 2018. World J Surg. (2019) 43:659–95. doi: 10.1007/s00268-018-4844-y, PMID: 30426190

[35] Fang L Zhang H. Application of rapid rehabilitation concept in gynecological single-hole laparoscopic surgery nursing. China Continuing Med Education. (2019) 12:171–4.

[36] Aiqing Wu. Rapid recovery in gynecological surgery surgical principle and application effect of anesthesia management research. J baotou Med college. (2019) 35 :17–20.

[37] Stein MJ Frank SG Lui A Zhang T Zhang. Ambulatory latissimus dorsi flap breast reconstruction: A prospective cohort study of an enhanced recovery after surgery (ERAS) protocol. J Plast Reconstr Aesthet Surg. (2019) 72:1950–5. doi: 10.1016/j.bjps.2019.06.039, PMID: 31488381

[38] Bisch SP Jago CA Kalogera E Ganshorn H Meyer LA Ramirez PT. Outcomes of enhanced recovery after surgery (ERAS) in gynecologic oncology-A systematic review and meta-analysis. Gynecol Oncol. (2021) 161:46–55. doi: 10.1016/j.ygyno.2020.12.035, PMID: 33388155

[39] Amjad T Malana MA Khan MSN Hasan SA Fahad S Haider M. Optimizing surgical outcomes: the role of enhanced recovery after surgery (ERAS) protocols in improving recovery and reducing hospital stays in Pakistan. Cureus. (2025) 17:e76713. doi: 10.7759/cureus.76713, PMID: 39897315 PMC11783200

[40] Jin O Xu T Lai J He J Wu Y Yang X. Impact of enhanced recovery after surgery concept process optimization on the perioperative period of gynecologic laparoscopic surgery. BMC Womens Health. (2025) 25:120. doi: 10.1186/s12905-025-03626-1, PMID: 40087739 PMC11907852

[41] Slavchev S Yordanov A. Enhanced Recovery After Surgery (ERAS) protocol in minimally invasive gynecological surgery: a review of the literature. Pol Przegl Chir. (2022) 95:1–5. doi: 10.5604/01.3001.0015.8687, PMID: 36805992

[42] Peters A Siripong N Wang L. Enhanced recovery after surgery outcomes in minimally invasive nonhysterectomy gynecologic procedures. Am J Obstet Gynecol. (2020) 223:234.e1–8., PMID: 32087147 10.1016/j.ajog.2020.02.008 PMC7395891

[43] Jenkins ES Crooks R Sauro K Nelson G. Enhanced recovery after surgery (ERAS) guided gynecologic/oncology surgery - The patient’s perspective. Gynecol Oncol Rep. (2024) 55:101510. doi: 10.1016/j.gore.2024.101510, PMID: 39323937 PMC11422566

[44] Radha M Donimath K Harshitha H Kurdi M Theerth K. Outcomes of enhanced recovery after surgery (ERAS) protocol implementation in major gynecologic procedures: A prospective case-control study. Cureus. (2025) 17:e84335. doi: 10.7759/cureus.84335, PMID: 40535390 PMC12174707

[45] Bizzarri N Chiantera V Ercoli A Fagotti A Tortorella L Conte C. Minimally invasive pelvic exenteration for gynecologic Malignancies: A multi-institutional case series and review of the literature. J Minim Invasive Gynecol. (2019) 26:1316–26., PMID: 30611973 10.1016/j.jmig.2018.12.019

[46] Gueli Alletti S Capozzi VA Rosati A De Blasis I Cianci S Vizzielli G. Laparoscopy vs. laparotomy for advanced ovarian cancer: a systematic review of the literature. Minerva Med. (2019) 110:341–57., PMID: 31124636 10.23736/S0026-4806.19.06132-9

